# CircRNA circ-NNT mediates myocardial ischemia/reperfusion injury through activating pyroptosis by sponging miR-33a-5p and regulating USP46 expression

**DOI:** 10.1038/s41420-021-00706-7

**Published:** 2021-11-29

**Authors:** Xiaomiao Ye, Yanwen Hang, Yi Lu, Dandan Li, Fangfang Shen, Ping Guan, Jian Dong, Ludong Shi, Wei Hu

**Affiliations:** Department of Cardiology, Minhang Hospital, Fudan University, Shanghai, China

**Keywords:** Heart failure, miRNAs

## Abstract

Pyroptosis has been implicated in the pathophysiology of myocardial infarction (MI) in rodents, but its contribution to reperfusion injury in MI patients is unclear. Here, we evaluated pyroptosis in MI patients in vitro and in vivo models of myocardial ischemia/reperfusion (I/R) injury. We also investigated the molecular mechanisms that regulate pyroptosis and myocardial I/R injury in these in vitro and in vivo models. The study showed that MI patients exhibited elevated serum concentrations of the pyroptosis-related pro-inflammatory cytokines IL-1β and IL-18. Increased levels of IL-1β and IL-18 as well as the pyroptosis-related inflammatory caspases (caspase-1 and 11) were detected in cultured cardiomyocytes after anoxia/reoxygenation (A/R) and in cardiac tissues after I/R. Circ-NNT and USP46 were upregulated while miR-33a-5p was downregulated in MI patients, as well as in cultured cardiomyocytes after A/R and cardiac tissues after I/R. Circ-NNT or USP46 knockdown or miR-33a-5p overexpression inhibited the expression of pro-caspase-1, cleaved caspase-1, pro-caspase-11, cleaved caspase-11, IL-1β, and IL-18 in A/R cardiomyocytes and attenuated myocardial infarction in I/R mice. The results from luciferase reporter assays and gene overexpression/knockdown studies indicated that miR-33a-5p directly targets USP46, and circ-NNT regulates USP46 by acting as a miR-33a-5p sponge. Direct association between circ-NNT and miR-33a-5p in cardiomyocytes was confirmed by pull-down assays. In summary, pyroptosis is activated during myocardial I/R and contributes to reperfusion injury. Circ-NNT promotes pyroptosis and myocardial I/R injury by acting as a miR-33a-5p sponge to regulate USP46. This circ-NNT→miR-33a-5p→USP46 signaling axis may serve as a potential target for the development of cardio-protective agents to improve the clinical outcome of reperfusion therapy.

## Introduction

Myocardial infarction (MI) is a major cause of death worldwide [1]. Rapid restoration of blood flow improves prognosis and survival; however, reperfusion after a period of ischemia frequently results in additional myocardial damage. Apoptosis, inflammation and oxidative injury are well-recognized mechanisms contributing to myocardial ischemia/reperfusion (I/R) injury [2–4]. Infiltration of monocytes, leukocytes, and other inflammatory cells occurs soon after ischemia [5], and post-I/R inflammation has been shown to aggravate myocardial damage [6]. Hence, cell death forms other than immunologically silent apoptosis are likely involved in the pathogenesis of myocardial I/R injury.

Pyroptosis is a form of pro-inflammatory programmed cell death mediated by the inflammatory caspases-1 (human and mouse) and caspases-11 (mouse) [7]. Inflammasome complexes are assembled in response to pathogens or altered-self signals. These inflammasomes convert pro-caspase-1 into the catalytically active caspase-1, which subsequently cleaves the proforms of interleukin (IL)-1β and IL-18 into their active forms. Unlike apoptosis, pyroptosis results in cell lysis and release of inflammatory cytokines to the extracellular space [8]. Pyroptosis serves as a host defense mechanism against microbial infections [9]. In addition, because of its pro-inflammatory features, pyroptosis contributes to pathogenesis of inflammatory and autoimmune diseases such as systemic lupus erythematosus [10], atherosclerosis [11] and I/R-induced acute kidney failure [12]. With regard to MI, increased levels of the NLRP3 inflammasome, IL-1β, and IL-18 were detected in cardiomyocytes and cardiac fibroblasts in mouse hearts after I/R, and NLRP3-deficient mouse hearts were protected from I/R injury [13]. In addition, VX-765, a highly selective caspase-1 inhibitor reduced infarct size to a similar extent as ischemic preconditioning in a rat model of MI [14]. Nonetheless, the clinical significance of pyroptosis in MI has not been established, and the factors controlling this form of pro-inflammatory cell death are not fully understood.

Ubiquitination, a highly conserved mechanism, is a crucial method of protein degradation in which proteins to be degraded are clearly labelled with E3 ubiquitin ligases and are transferred to lysosomes for degradation [15]. Further, this process is reversible by deubiquitinating enzymes (DUBs) such as ubiquitin-specific protease-46 (USP46), which removes the label/ubiquitin thus preventing its degradation [16]. Relatively less is known about this class of proteins, and the available information on the roles of USP46 is varied and not abundant. For example, a study on colon cancer [17] indicates USP46 is a tumor suppressor whereas in human papilloma virus transformed cancers [18], USP46 is necessary for the proliferation of the tumor cells. Therefore, there is a need for elaborate studies specifically in relation to its role in myocardial infarction.

Circular RNAs (or circRNAs) are a type of non-coding RNAs in which the 3′ and 5′ ends normally present in an RNA molecule have been joined together [19]. CircRNAs are usually expressed at low levels and exhibit tissue-specific expression patterns [20]. Studies have shown that circRNAs can regulate gene expression by acting as microRNA (miRNA) and protein sponges, and by modulating RNA polymerase II transcription and pre-mRNA processing [19]. Emerging evidence has implicated circRNAs in diseases in the central nervous and cardiovascular systems, as well as in cancer [21], but the function of circRNAs in regulating pyroptosis and MI is unclear. In this study, we evaluated pyroptosis in MI patients and in in vitro and in vivo models of myocardial I/R injury. We also investigated the function of circRNA hsa_circ_0072424 (named as circ-NNT) as a regulator of pyroptosis and myocardial I/R injury, as well as the underlying mechanisms involving miR-33a-5p and USP46.

## Results

### Pyroptosis is activated following myocardial I/R in vivo

**Fig. 1:**
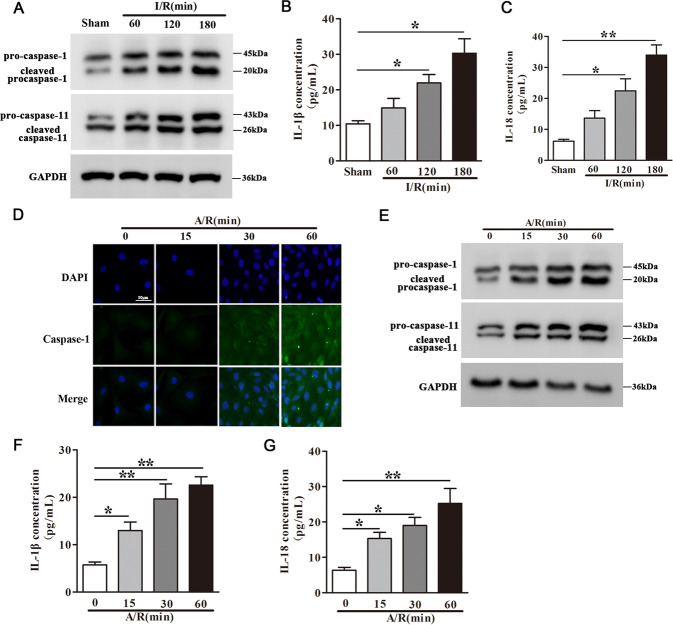
Pyroptosis is activated following myocardial I/R in vivo and in cultured cardiomyocytes following A/R in vitro. In vivo, C57BL/6 mice were subjected to 45 min myocardial ischemia followed by 60, 120, 180 min reperfusion as indicated. The sham group was included as control **A**–**C**. **A** Relative protein expression of pro-caspase-1, cleaved caspase-1, pro-caspase-11, and cleaved caspase-11 in heart tissues were determined by western blot analysis. **B**, **C** The concentrations of IL-1β (**B**) and IL-18 (**C**) were detected by ELISA assay. *n* = 6. **D**–**G** In vitro, Cardiomyocytes were subjected to 30 min anoxia followed by 0, 15, 30, 60 min reoxygenation. **D** Immunofluorescence staining for caspase-1 (magnification ×200) was determined. The cells were stained with DAPI (caspase-1: green; nucleus: blue). **E** The relative protein expression of pro-caspase-1, cleaved caspase-1, pro-caspase-11, and cleaved caspase-11 were determined by western blot analysis. **F**, **G** The relative concentrations of IL-1β (**F**) and IL-18 (**G**) were detected by ELISA assay, *n* = 3. *P < 0.05, **P < 0.01, ***P < 0.001.

Compared with sham group, mice exposed to 45 min myocardial ischemia followed by 60, 120 or 180 min reperfusion showed significantly higher pro-caspase-1, cleaved caspase-1, pro-caspase-11 and cleaved caspase-11 protein expression in the heart, and the expression increased with reperfusion time (Fig. 1A). Pro-caspase-1, cleaved caspase-1, pro-caspase-11 and cleaved caspase-11 mRNA expression after I/R exhibited a similar pattern to that of the proteins (Fig. S1A, B). Moreover, elevated cardiac mRNA and the concentrations of IL-1β and IL-18 were detected after I/R (Fig. 1B, C and Fig. S1C, D), these results indicated the activation of pyroptosis following myocardial I/R in these animals.

### Pyroptosis is activated in cultured cardiomyocytes following anoxia/reoxygenation (A/R)

To evaluate pyroptosis in an in vitro model of cardiomyocytes following anoxia/reoxygenation (A/R), we exposed cultured mouse cardiomyocytes to 30 min anoxia followed by 0, 15, 30, and 60 min reoxygenation. Cell immunofluorescence revealed time-dependent increases in caspase-1 staining within the 0, 15, 30, and 60 min reoxygenation (Fig. 1D). Subsequent RT-PCR and western blot analysis showed time-dependent increases in pro-caspase-1, cleaved caspase-1, pro-caspase-11, and cleaved caspase-11 expressions at both mRNA and protein levels (Fig. 1E and Fig. S1E, F). Similar increases in both IL-1β and IL-18 mRNA expression (Fig. S1G, H) and protein concentration into the culture medium were found (Fig. 1F, G). These data indicated that A/R treatment activates pyroptosis in cardiomyocytes, and the extent of pyroptosis grows with reoxygenation time.

### CircRNA profiles in the myocardial sham group and I/R group in mice

**Fig. 2:**
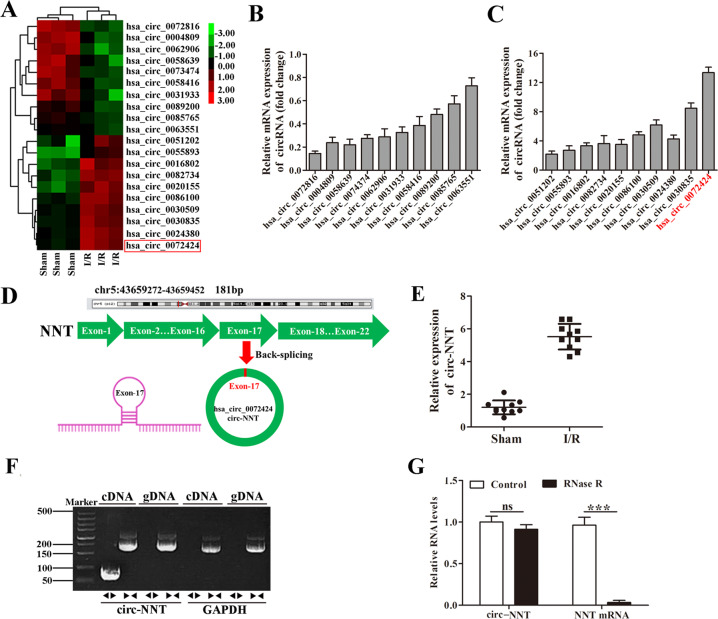
Expression profiles of circRNAs in sham group and myocardial I/R group in vivo. **A** The heat map shows the 20 differently expressed circRNAs, which were analyzed by circRNAs Arraystar Chip. **B** The relative mRNA expression of 10 down-regulated circRNAs were detected by qRT-PCR (*n* = 10/group). **C** The relative mRNA expression of 10 up-regulated circRNAs were detected by qRT-PCR (*n* = 10/group). **D** Scheme illustrating the production of circ-NNT. Circ-NNT was formed by back splicing at exon 17 of the NNT gene. **E** The relative mRNA expression of circ-NNT was shown in a scatter plots (the top upregulated circRNAs), which was measured by qRT-PCR (*n* = 10/group). **F** The gel electrophoresis validated the existence of circ-NNT. **G** Circ-NNT and NNT mRNA levels in cardiomyocytes with or without RNase R treatment were measured by qRT-PCR. ns: no significant, ***P < 0.0001.

Using a circRNA microarray technique, we evaluated the circRNA profiles of sham group and myocardial I/R group in mice. The results of hierarchical clustering show a distinct circRNA expression profiling between the two groups (Fig. 2A). Then we examined the relative mRNA expression of the 20 circRNAs between the sham group and myocardial I/R group to confirm their expression, the 20 up-regulated and down-regulated circRNAs were detected by qRT-PCR (Fig. 2B, C). In the results, we found that the hsa_circ_0072424 (circ-NNT) were most differently expressed. Then in silico analysis predicted that circ-NNT was composed of 181 bp and derived from exon 17 of the NNT gene (Fig. 2D). Thus, we focused on the expression and role of circ-NNT in the progression of myocardial I/R injury in this study. The relative expression of circ-NNT was detected by qRT-PCR (Fig. 2E), the results indicated that the circ-NNT was significantly increased in I/R group compared to the sham group. To detect whether the head-to tail splicing of circ-NNT results from trans-splicing or genomic rearrangements, we extracted cDNA and gDNA from cardiomyocytes. The gel electrophoresis results showed that circ-NNT was detected only in cDNA, but not gDNA, indicating that the loop structure of circ-NNT comes from the reverse splicing (Fig. 2F). To check for circularity of circ-NNT, cardiomyocytes were treated with RNase R, the results showed that circ-NNT was more resistant to RNase R treatment compared to mRNA NNT (Fig. 2G). In addition, we measured the abundance of circ-NNT in various tissues following myocardial ischemia/reperfusion injury. The results showed that circ-NNT was predominantly expressed in the heart, suggesting the circ-NNT is a well expressed heart- specific tissue circRNA (Fig. S2). Through UCSC database (http://genome.ucsc.edu), we identified NNT as a nearby gene for circ-NNT (Fig. S3A). And then, conservative analysis of the full length sequence in 7 different species was conducted by UCSC database, the full length sequence of circ-NNT was blasted against the human circ-NNT in circBase (http://www.circbase.org/), circ-NNT (hsa_circ_0072424) was found to have high homology (Fig. S3B). These results suggest that the biogenesis of circ-NNT is conserved in humans.

### Circ-NNT is induced by I/R and mediates myocardial I/R injury in vivo

**Fig. 3:**
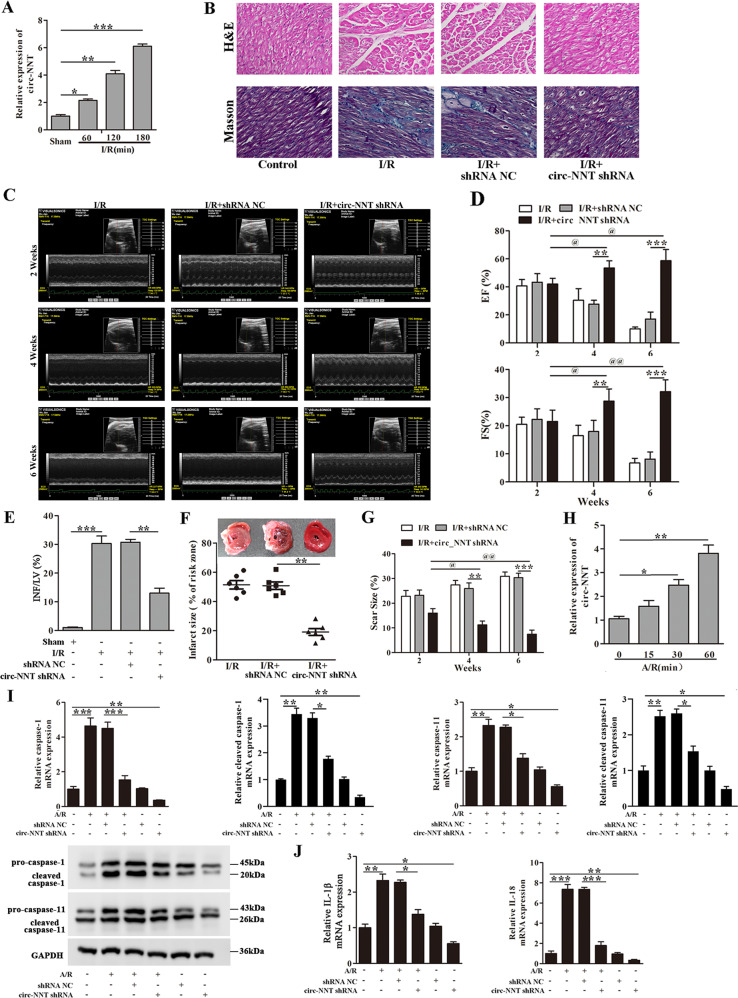
Circ-NNT is induced by I/R and mediates myocardial I/R injury in vivo and A/R-stimulated pyroptosis in cardiomyocytes. **A** Mice were subjected to 45 min myocardial ischemia followed by 60, 120, or 180 min reperfusion. Sham mice were included as control. Relative circ-NNT expression in cardiac tissues was determined by RT-PCR. *n* = 6/group. **B** Representative images of H&E and Masson trichrome staining of the LV sections were determined, *n* = 6. Cardiomyocytes were subjected to 30 min anoxia followed by 0, 15, 30, or 60 min reoxygenation. **C** Representative M-mode echocardiography of mice at 2, 4, and 6 weeks were detected after myocardial I/R. Mice were treated with shRNA NC or circ-NNT shRNA. **D** The percentages of Ejection Fraction (EF%) and Fractional shortening (FS%) were measured at 2, 4, and 6 weeks after myocardial I/R. **E**, **F** Mice received adenoviruses carrying circ-NNT shRNA or shRNA NC by injection as described in methods. After 5 days, the mice were subjected to myocardial I/R (45 min/180 min). INF/LV (%) and Infract size (% of risk zone) are shown. **G** Scar size at 2, 4, and 6 weeks of left ventricle was determined after myocardial I/R. **H** Relative circ-NNT levels were determined by RT-PCR. *n* = 3. Cardiomyocytes transfected with circ-NNT shRNA or shRNA NC were subjected to A/R (30 min/60 min) as indicated. **I** Relative protein and mRNA expression of pro-caspase-1, cleaved caspase-1, pro-caspase-11 and cleaved caspase-11 were determined by western blot analysis and RT-PCR, respectively. **J** Relative mRNA expression of IL-1β and IL-18 were detected by RT-PCR. *n* = 3. *P < 0.05, **P < 0.01, ***P < 0.001.

To evaluate circ-NNT function in myocardial I/R injury in vivo, we determined the mRNA expression of circ-NNT, the results indicate that circ-NNT was upregulated in cardiac tissues after I/R and the expression levels increased with reperfusion time (Fig. 3A). To reveal the function of circ-NNT in vivo, we tested the effects of circ-NNT knockdown by intracoronary delivery of adenoviruses carrying circ-NNT shRNA. Circ-NNT knockdown in I/R mice was confirmed by RT-PCR (Fig. S4A). Compared with sham, I/R mice exhibited marked cardiac structural abnormalities, mainly manifested in cardiomyocytes hypertrophy and interstitial fibrosis as revealed by H&E and Masson trichrome staining (Fig. 3B). The results demonstrate that these I/R-induced abnormalities were attenuated by circ-NNT knockdown. At 2, 4 and 6 weeks post I/R, the mice exhibited a pronounced decline in LV function, reflected by an attenuation of LV wall motion and reduction of EF% and FS%. Further, circ-NNT knockdown in I/R mice alleviated the decline of LV function and increase EF% and FS% (Fig. 3C, D). In I/R groups, the myocardial fiber arrangement was disordered, myocardial cell degeneration was mild, fibrosis was severe, inflammatory cell infiltration and gap widening were increased with the increasing treatment time. Circ-NNT knockdown alleviated myocardial fibrosis and myocardial cell degeneration and decreased gap widening (Fig. S5A). Hence, the LV function was significantly improved by circ-NNT knockdown in I/R mice. Moreover, circ-NNT knockdown I/R mice displayed reduced INF/LV and infract size compared with shRNA NC-treated I/R mice (Fig. 3E, F). Moreover, a planimetric analysis showed a slight increase in scar size of the left ventricle in I/R mice. Circ-NNT knockdown could significantly reduce the scar size with the increase of treatment time, and the scar size was reduced from 31.37% to 9.09% at 6 weeks (Fig. 3G). Collectively, these data indicated that circ-NNT contributes to I/R-induced cardiac injury and dysfunction in vivo.

### Circ-NNT mediates A/R-stimulated pyroptosis in cultured cardiomyocytes

To evaluate the expression of circ-NNT in cardiomyocytes, the RT-PCR results indicated that circ-NNT was upregulated in cultured cardiomyocytes after A/R and the expression levels increased with reoxygenation time (Fig. 3H). We subsequently tested the effects of circ-NNT knockdown in cardiomyocytes by adenoviral delivery of circ-NNT shRNA. Circ-NNT knockdown in cardiomyocytes was confirmed by RT-PCR before and after A/R treatment (Fig. S4B, C). Cell immunofluorescence revealed lower caspase-1 expression after A/R in circ-NNT knockdown cardiomyocytes compared with control (Fig. S4D). In addition, western blot and RT-PCR data showed lower levels of pro-caspase-1, cleaved caspase-1, pro-caspase-11, cleaved caspase-11, IL-1β, and IL-18 in circ-NNT knockdown cardiomyocytes after A/R treatment compared with control (Fig. 3I, J). Thus, circ-NNT positively regulates cardiomyocytes pyroptosis in vitro.

### Circ-NNT directly binds to miR-33a-5p in cardiomyocytes

**Fig. 4:**
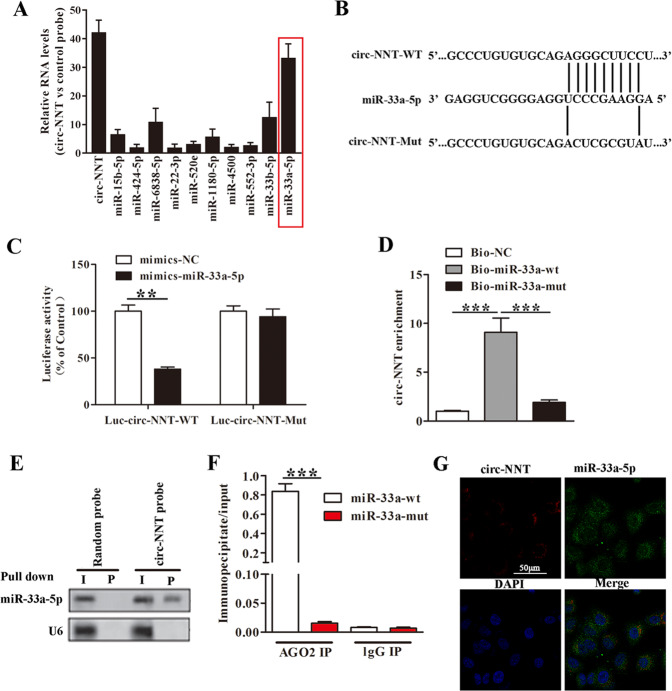
Circ-NNT directly binds to miR-33a-5p in cardiomyocytes. **A** Sequences of circ-NNT segment containing the putative miR-33a-5p binding site (circ-NNT-WT) and a mutant circ-NNT segment (circ-NNT-Mut) with mutations at the putative miR-33a-5p binding site are shown. **B** Cardiomyocytes were transfected with mimics-miR-33a-5p/mimics-NC along with the luciferase construct carrying circ-NNT-WT or circ-NNT-Mut as indicated. The luciferase activity was detected after 48 h transfection, *n* = 3. **C** circRNA immunoprecipitation assays were performed in circ-NNT-overexpressing 293 T cells using a circ-NNT-specific probe and control probe, respectively. The enrichment of circ-NNT and microRNAs was detected by qRT-PCR and normalized to the control probe. **D** Cardiomyocytes were transfected with Bio-miR-33a-5p-WT, Bio-miR-33a-5p-Mut or Bio-NC. RNAs bound to the biotinylated probe were collected by biotin-based pull down assays, and the circ-NNT enrichment was detected by RT-PCR, *n* = 3. **E** Cardiomyocytes lysates were incubated with circ-NNT probe or random probe-coated magnetic beads. RNAs bound to the beads were extracted, and miR-33a-5p was detected by northern blot analysis. I, input (10% samples were loaded); P, pellet (100% samples were loaded). **F** Cardiomyocytes were transfected with miR-33a-5p-WT or miR-33a-5p-Mut. RNAs bound to anti-AGO2 antibody or IgG-coated sepharose beads were collected by AGO2 immunoprecipitation assays, and circ-NNT was detected by RT-PCR. The immunoprecipitate/input ratios are shown, *n* = 5. **G** Cardiomyocytes were co-transfected with circ-NNT and miR-33a-5p. Co-localization of circ-NNT and miR-33a-5p were visualized by situ hybridization. Cells were stained with DAPI. *P < 0.05, **P < 0.01, ***P < 0.001.

To further understand circ-NNT’s various binding targets; we conducted immunoprecipitation studies in circ-NNT overexpressing HEK293T cells using a probe specific to circ-NNT and a control probe, respectively (Fig. 4A). The putative candidate miRNAs binding to circ-NNT were predicted using StarBase (http://starbase.sysu.edu.cn/mirMrna.php). The enrichment of circ-NNT and microRNAs (miRNAs) was detected by qRT-PCR and normalized to the control probe. Based on the evidence, it was clear that many miRNAs were enriched with circ-NNT. Among these miR-33a was most highly expressed, hence we considered this as our potential candidate. To investigate the relationship between circ-NNT and miR-33a-5p, we prepared luciferase reporter constructs carrying a circ-NNT segment containing the putative miR-33a-5p binding site (Luc-circ-NNT-WT) or a corresponding mutant circ-NNT segment (Luc-circ-NNT-Mut) with mutations at the putative binding site (Fig. 4B). Co-transfection with mimics-miR-33a-5p inhibited the luciferase activity in Luc-circ-NNT-WT but not in Luc-circ-NNT-Mut-transfected cardiomyocytes (Fig. 4C).

Further, we tested whether circ-NNT could directly bind to miR-33a-5p in vivo. We applied a biotin-avidin pull down system to test whether miR-33a-5p could pull down circ-NNT. In the biotin-miRNA-based pull down assay, circ-NNT was detected at remarkably higher levels in RNA complexes pulled down by Bio-miR-33a-5p-WT-coated beads than those by Bio-miR-33a-5p-Mut or Bio-NC-coated beads (Fig. 4D). Circ-NNT was pulled down and analysed by qRT-PCR, the introduction of mutations that disrupt base pairing between circ-NNT and miR-33a-5p led to the inability of miR-33a-5p to pull down circ-NNT, indicating that miR-33a-5p recognizes circ-NNT in a sequence-specific manner. Moreover, we also employed inverse pull-down assay to test whether circ-NNT could pull down miR-33a-5p using biotin-labelled specific circ-NNT probe. MiR-33a-5p was co-precipitated and analyzed by northern blot (Fig. 4E). In the AGO2 immunoprecipitation assay, AGO2 immunoprecipitates from mimics-miR-33a-5p-transfected cardiomyocytes showed significantly higher circ-NNT contents compared with those from miR-33a-5p-Mut-transfected cells (Fig. 4F). These data provided solid evidence for direct association between circ-NNT and miR-33a-5p. In line with this, RNA in situ hybridization revealed co-localization of circ-NNT and miR-33a-5p in cardiomyocytes (Fig. 4G).

### MiR-33a-5p protects against pyroptosis and myocardial I/R injury

**Fig. 5:**
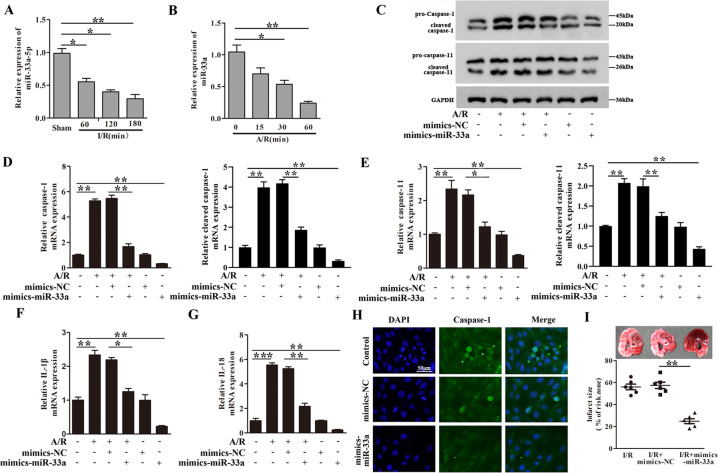
MiR-33a-5p protects against pyroptosis and myocardial I/R injury by downregulating USP46. **A** Mice were subjected to 45 min myocardial ischemia followed by 60, 120, and 180 min reperfusion. Relative miR-33a-5p levels in cardiac tissues were determined by RT-PCR. Sham mice were included as control. *n* = 6. **B** Cardiomyocytes were subjected to 30 min anoxia followed by 0, 15, 30, and 60 min reoxygenation. Relative miR-33a-5p levels were determined by RT-PCR, *n* = 3. **C**–**H** Cardiomyocytes transfected with mimics-miR-33a-5p or mimics-NC were subjected to A/R (30 min/60 min). **C**–**E** Relative protein and mRNA levels of pro-caspase-1, cleaved caspase-1, pro-caspase-11, and cleaved caspase-11 were detected by western blot analysis and RT-PCR, respectively. **F**, **G** Relative mRNA levels of IL-1β (**F**) and IL-18 (**G**) were determined by RT-PCR. **H** Immunofluorescence staining for caspase-1 (magnification ×200) was determined. The cells were stained with DAPI, *n* = 3. **I** Mice received mimics-miR-33a-5p/mimics-NC and adenoviruses carrying pcDNA-USP46 or pcDNA empty vector as described in methods, alone or in combination as indicated. After 5 days, the mice were subjected to myocardial I/R (45 min/180 min). INF/LV (%) is shown, *n* = 6. *P < 0.05, **P < 0.01, ***P < 0.001.

In contrast to circ-NNT, miR-33a-5p was down-regulated in mice after I/R and in cardiomyocytes after A/R (Fig. 5A, B). MiR-33a-5p mimics inhibited A/R-stimulated pyroptosis of cardiomyocytes as reflected in the decreased expression of pro-caspase-1, cleaved caspase-1, pro-caspase-11, cleaved caspase-11, IL-1β and IL-18 (Fig. 5C–G). Cell immunofluorescence revealed that miR-33a-5p mimics decreased caspase-1 expression in A/R-treated cardiomyocytes (Fig. 5H). Moreover, miR-33a-5p mimics attenuated myocardial I/R injury in mice as indicated in reduced infract size (Fig. 5I), indicating that miR-33a-5p protects against myocardial I/R injury.

### MiR-33a-5p directly regulates USP46

**Fig. 6:**
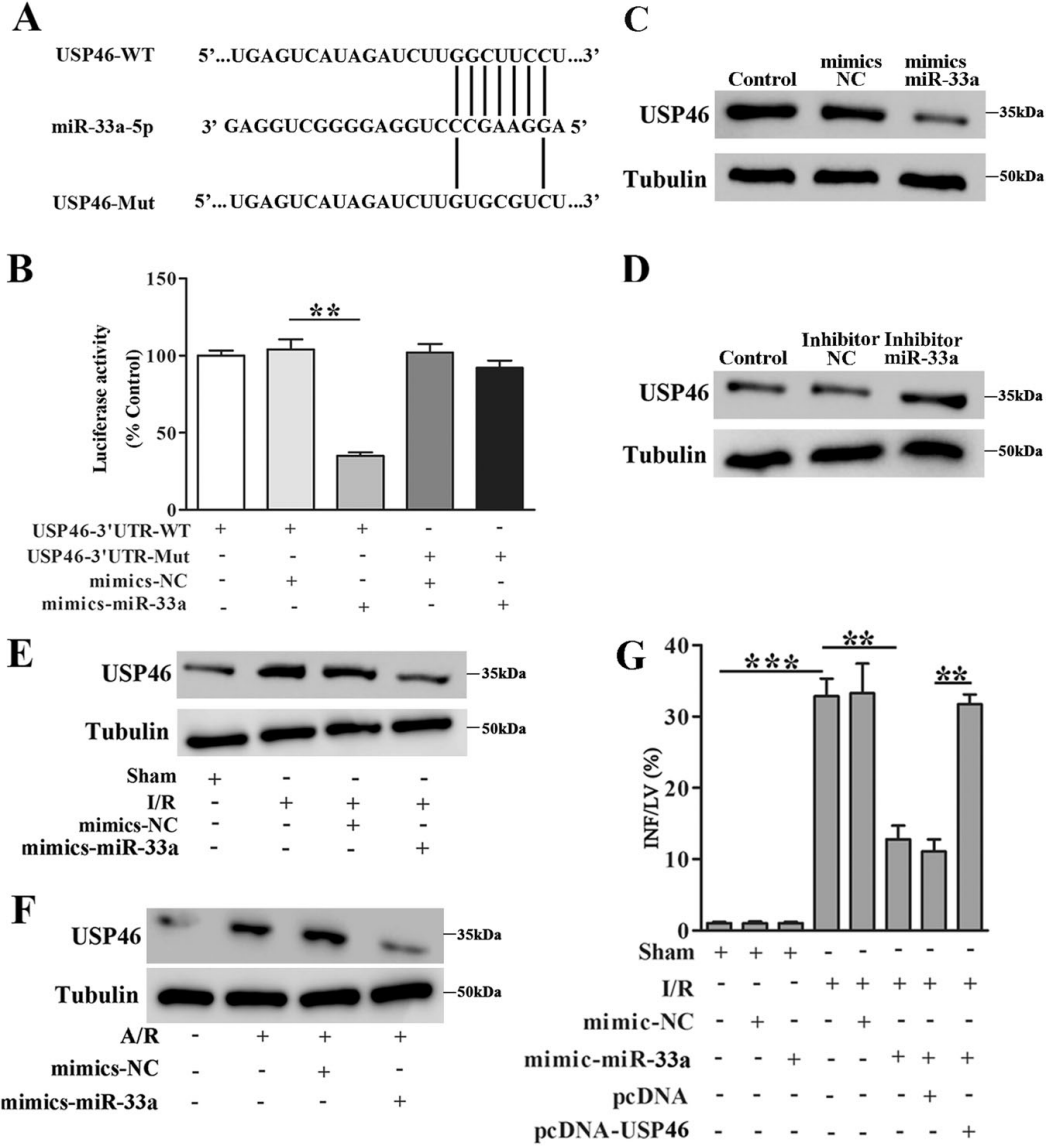
MiR-33a-5p directly regulates USP46. **A** Sequences of USP46 3′-UTR (USP46-WT) and a mutant USP46 3′-UTR derivative (USP46-Mut) with mutations at the putative miR-33a-5p binding site are shown. **B** HEK293 cells were transfected with mimics-miR-33a-5p/mimics-NC along with the luciferase construct carrying USP46-WT or USP46-Mut as indicated. The luciferase activity was detected after 48 h transfection, *n* = 3. **C**, **D** USP46 expression was determined by western blot analysis. Cardiomyocytes were transfected with mimics-miR-33a-5p/mimics-NC (**C**) or inhibitor-miR-33a-5p/inhibitor-NC (**D**). **E** Mice received mimics-miR-33a-5p or mimics-NC by injection as described in methods, and were subsequently subjected to myocardial I/R (45 min/180 min). USP46 levels in cardiac tissues were determined by western blot analysis. **F** Cardiomyocytes transfected with mimics-miR-33a-5p or mimics-NC were subjected to A/R (30 min/60 min). USP46 levels were determined by western blot analysis. **G** Mice received mimics-miR-33a-5p or mimics-NC as described in methods and subsequently subjected to myocardial I/R (45 min/180 min). Infarct size (% of risk zone) is shown, *n* = 6. **P < 0.01, ***P < 0.001.

The results from the luciferase reporter assay showed that co-transfection with mimics-miR-33a-5p inhibited luciferase activity in Luc-USP46-3’UTR-WT but not in Luc-USP46-3’UTR-Mut-transfected cells (Fig. 6A, B). Collectively, these data indicated that miR-33a-5p directly targets USP46 by binding to USP46-3’UTR. To investigate the relationship between miR-33a-5p and USP46, we studied the effects of miR-33a-5p mimics and inhibitor. Mimics-miR-33a-5p transfection decreased while miR-33a-5p inhibitor transfection increased USP46 mRNA expression in cardiomyocytes (Fig. 6C, D). Mimics-miR-33a-5p transfection also inhibited USP46 expression stimulated by myocardial I/R injury in vivo and A/R in cardiomyocytes in vitro (Fig. 6E, F). In addition, USP46 overexpression promoted myocardial infarction, and we found that USP46 restored myocardial infarction inhibited by miR-33a-5p mimics (Fig. 6G), indicating that miR-33a-5p protects against myocardial I/R injury through down-regulation of USP46.

### USP46 mediates cardiomyocytes pyroptosis in vitro and myocardial I/R injury in vivo

**Fig. 7:**
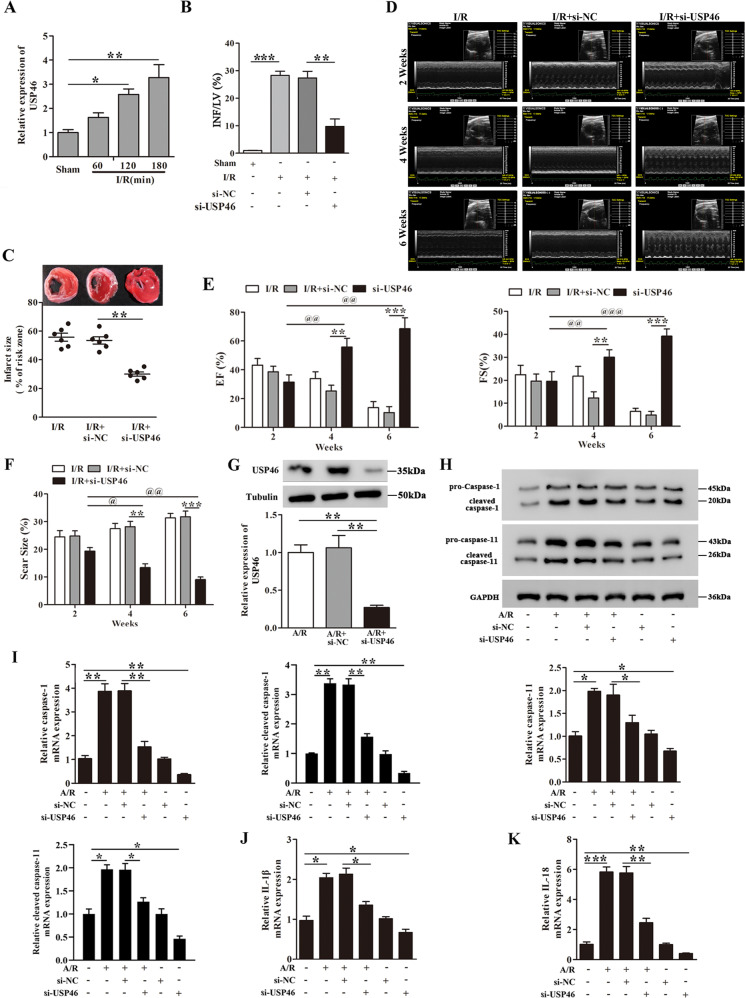
USP46 mediates cardiomyocytes pyroptosis in vitro and myocardial I/R injury in vivo. Mice were subjected to 45 min myocardial ischemia followed by 60, 120, or 180 min reperfusion. **A** Relative USP46 mRNA levels in cardiac tissues were determined by RT-PCR. *n* = 6. **B**, **C** Mice received adenoviruses carrying si-USP46 or si-NC by injection as described in methods. After 5 days, the mice were subjected to myocardial I/R (45 min/180 min). INF/LV (%) and Infract size (% of risk zone) are detected. *n* = 6. **D** Representative M-mode echocardiography at 2, 4, and 6 weeks after myocardial I/R were examined. Mice were treated with si-NC or si-USP46 (*n* = 6). **E** The percentages of Ejection Fraction (EF%) and Fractional shortening (FS%) were measured at 2, 4, and 6 weeks after myocardial I/R. **F** Scar size at 2, 4, and 6 weeks of left ventricle were determined after myocardial I/R. **G** Relative protein and mRNA expression of USP46 in cardiomyocytes were detected by western blot analysis and RT-PCR, respectively. Untransfected cells were included as control. *n* = 3. Cardiomyocytes transfected with si-USP46 or si-NC were subjected to A/R (30 min/60 min). **H**, **I** Relative protein and mRNA levels of pro-caspase-1, cleaved caspase-1, pro-caspase-11 and cleaved caspase-11 were detected by western blot and RT-PCR, respectively. **J**, **K** Relative mRNA levels of IL-1β (**J**) and IL-18 (**K**) were determined by RT-PCR, *n* = 3. *P < 0.05, **P < 0.01, ***P < 0.001.

We measured the expression of USP46 in various tissues following myocardial I/R injury. The results demonstrated that USP46 was mainly expressed in the heart, indicating the USP46 is a well-expressed heart-specific tissue (Fig. S2). Compared with sham group, mice subjected to myocardial I/R exhibited increased USP46 mRNA expression in the heart (Fig. 7A). USP46 knockdown by intracoronary delivery of adenoviruses carrying si-USP46 effectively ameliorated cardiac injury after I/R as indicated in reduced INF/LV and infarct size (Fig. 7B, C). At 2, 4, and 6 weeks post I/R, the mice exhibited a pronounced decline in LV function, reflected by a promotion of LV wall motion and reduction of EF% and FS%, USP46 knockdown in I/R mice alleviated the decline of LV function, and increase EF% and FS% (Fig. 7D, E). The results of H&E staining at 2, 4 and 6 weeks were shown in Fig. S5B. Compared with I/R groups, the myocardial fiber arrangement was disordered, myocardial cell degeneration was mild, fibrosis was severe, inflammatory cell infiltration and gap widening were increased. USP46 knockdown relieved myocardial fibrosis and myocardial cell degeneration and decreased gap widening with the increasing treatment time. Hence, the LV function was significantly improved by USP46 knockdown in I/R mice. A planimetric analysis showed a slight increase of scar size of the left ventricle in I/R mice. USP46 knockdown could significantly reduce the scar size from 31.37% to 9.09% at 6 weeks (Fig. 7F). USP46 knockdown by si-USP46 transfection was confirmed by both RT-PCR and western blot analysis In A/R-treated cardiomyocytes (Fig. 7G). In cardiomyocytes, USP46 knockdown inhibited the expression of pro-caspase-1, cleaved caspase-1, pro-caspase-11, cleaved caspase-11, IL-1β, and IL-18 induced by A/R treatment (Fig. 7H–K), these results suggested that the cardio-protective effects of USP46 knockdown in vivo are mediated by inhibition of pyroptosis.

### Circ-NNT regulates cardiomyocytes pyroptosis through miR-33a-5p/USP46 pathway

**Fig. 8:**
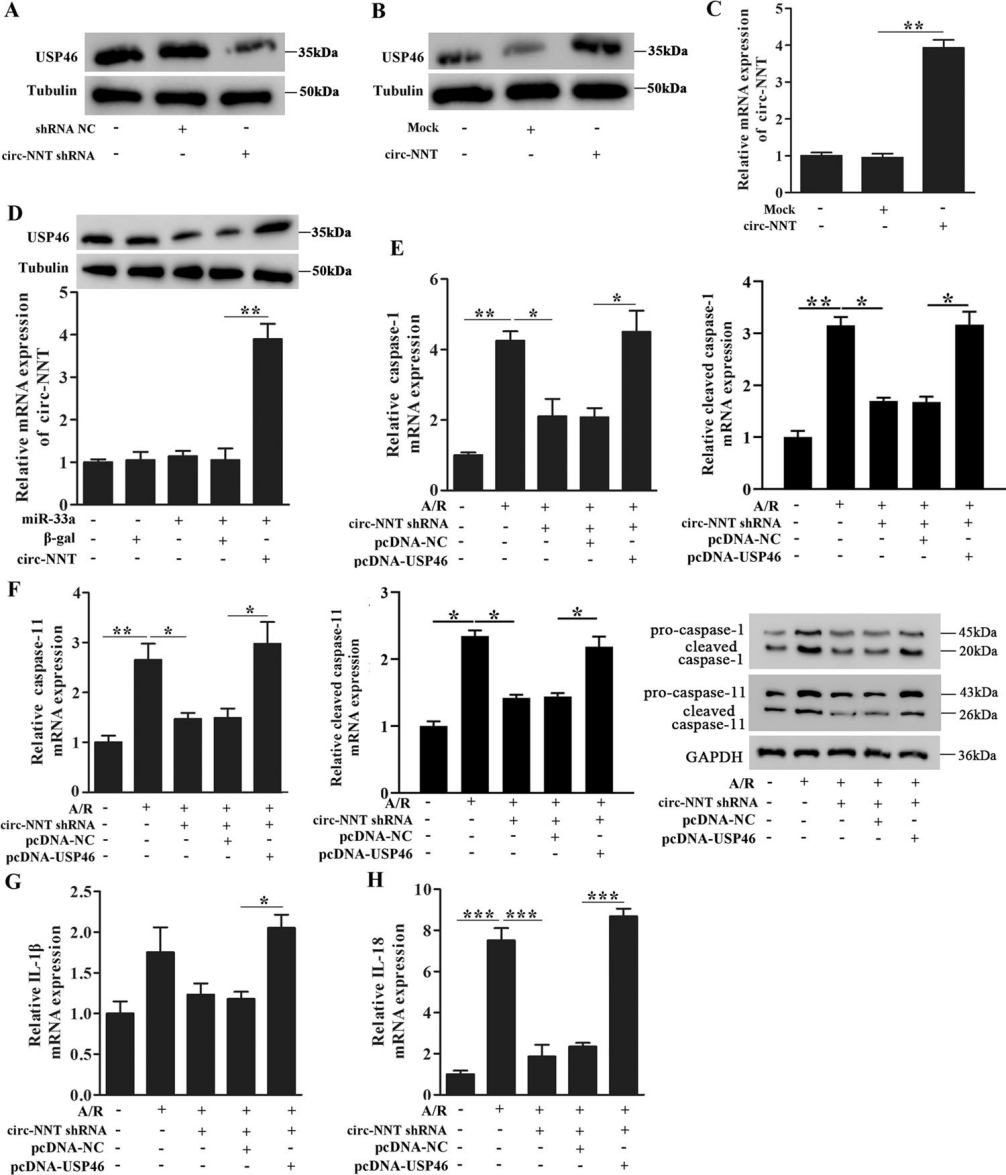
Circ-NNT mediates A/R-stimulated cardiomyocytes pyroptosis through miR-33a-5p and USP46. **A**, **B** USP46 protein expression was detected by western blot in cardiomyocytes, which were transfected with shRNA NC/circ-NNT shRNA or Mock/circ-NNT. **C** The relative circ-NNT expression was determined by RT-PCR in cardiomyocytes, which were transfected with Mock/circ-NNT. **D** Cardiomyocytes were transfected with miR-33a-5p, β-gal, and circ-NNT, alone or in combination as indicated. USP46 and circ-NNT levels were determined by western blot analysis and RT-PCR, respectively. **E**–**H** Cardiomyocytes transfected with circ-NNT shRNA, pcDNA-USP46, and pcDNA-NC, alone or in combination as indicated were subjected to A/R (30 min/60 min). **E**, **F** The protein and mRNA expression of pro-caspase-1, cleaved caspase-1, pro-caspase-11, and cleaved caspase-11 were detected by western blot and RT-PCR, respectively. **G**, **H** The mRNA expression of IL-1β (**G**) and IL-18 (**H**) were determined by RT-PCR. *n* = 3, *P < 0.05, **P < 0.01, ***P < 0.001.

Finally, we examined the relationship between circ-NNT and USP46. We found that circ-NNT knockdown inhibited USP46 expression while circ-NNT overexpression increased USP46 expression in cardiomyocytes (Fig. 8A–C). In addition, circ-NNT overexpression restored USP46 downregulated expression by mimics-miR-33a-5p (Fig. 8D). These data supported the circ-NNT → miR-33a-5p → USP46 regulatory axis we proposed at the beginning of this study. Finally, USP46 overexpression restored A/R-induced expression of pro-caspase-1, cleaved caspase-1, pro-caspase-11, cleaved caspase-11, IL-1β, and IL-18 in cardiomyocytes inhibited by circ-NNT knockdown (Fig. 8E–H), indicating that circ-NNT regulates cardiomyocytes pyroptosis through modulation of miR-33a-5p/USP46.

## Discussion

Despite significant medical advances, patients with acute MI suffer high mortality and morbidity [22, 23]. Although early and complete reperfusion effectively limits infarct size and cardiac remodeling, reperfusion by itself can cause irreversible injury to the myocardium and the coronary microvasculature, jeopardizing final patient outcome [24]. Numerous cardio-protective strategies on top of reperfusion have been explored, but most attempts have failed to reduce infarct size and improve clinical outcome [25]. At present, remote ischemic conditioning and a few drugs have been shown to reduce infarct size in early proof-of-concept clinical studies [26–29], but the therapeutic benefits still need to be confirmed in large clinical trials. Overall, reperfusion injury remains a daunting barrier to successful reperfusion therapy.

The infarcted myocardium exhibits morphological features of necrosis, which typically become more evident during reperfusion [30–32]. Unlike necrosis, which is considered an unregulated form of cell death, regulated forms of cell death with specific signal transduction mechanisms such as apoptosis, autophagy and necroptosis also occur in the infarcted myocardium [33]. Studies have shown that multiple types of cell death can occur simultaneously in response to the same stimulus [34, 35]. The mechanism of demise taken by individual cells is influenced by the local intensity of the insult and the type and activation/differentiation state of the cell per se [36, 37]. In addition, multiple cell death pathways may be activated simultaneously in a single dying cell, and the cross-talk between these programs determines the cell death process and the ultimate outcome [38]. Recognition of the different pathways of cardiomyocytes death during I/R encourages the development of novel cardio-protective strategies that target these processes.

Inflammation is a hallmark of I/R injury. A recent study implicated pyroptosis, the pro-inflammatory form of programmed cell death in rodent cardiomyocytes death during I/R, as elevated levels of the pyroptosis-related cytokines IL-1β and IL-18 were detected in the left ventricle after MI [13]. In line with these previous findings, we detected increased expression of IL-1β and IL-18 as well as the pyroptosis-related pro-inflammatory caspases (caspase-1 and caspase-11) in the myocardium after I/R in vivo and in cultured cardiomyocytes after A/R in vitro. In addition, we detected higher serum levels of IL-1β and IL-18 in I/R groups compared with sham groups, highlighting the significance of pyroptosis in the pathophysiology of MI in clinical settings. We subsequently investigated the mechanisms underlying the activation of pyroptosis in in vitro and in vivo models of myocardial I/R. Using bioinformatics analysis, we identified potential regulatory relationships between miR-33a-5p and USP46, and between circ-NNT and miR-33a-5p. All three genes were dysregulated in I/R groups, as indicated by microarray screening and confirmed by RT-PCR and ELISA. Our subsequent studies demonstrated that circ-NNT and USP46 positively regulate and miR-33a-5p negatively regulates A/R-induced cardiomyocytes pyroptosis in vitro and I/R-induced myocardial pyroptosis and infarction in vivo. The results from luciferase reporter assays and gene overexpression/knockdown studies indicated that miR-33a-5p directly targets USP44, and circ-NNT regulates USP46 by acting as a miR-33a-5p sponge. In line with this, direct association between circ-NNT and miR-33a-5p in cardiomyocytes was detected by multiple immunoprecipitation assays.

In summary, we identified a circ-NNT → miR-33a-5p → USP46 signaling axis that regulates cardiomyocytes pyroptosis and myocardial I/R injury. This signaling pathway may serve as a potential target for the development of cardio-protective agents to improve clinical outcome of reperfusion therapy. Circ-NNT may be an attractive target and a novel potential therapeutic strategy. Later, we will further study the function of circ-NNT→miR-33a-5p→USP46 pathway in rats and more other mammals in depth. Our ultimate goal is to achieve clinical diagnosis or treatment of circ-NNT.

## Materials and methods

### Clinical samples

Serum samples were collected from 25 patients with acute myocardial infarction and 25 healthy volunteers following the regulations of Minhang Central Hospital (Shanghai, China), this study is approved by Minhang Central Hospital, Fudan University. All study subjects provided written informed consent.

### Cardiomyocytes culture and treatment

Cardiomyocytes were isolated from male mice (1–2 days old) purchased from Minhang Central Hospital. Briefly, hearts were dissected, washed, and cut into small pieces in HEPES-buffered saline (HBS). The tissues were then digested with pancreatin (1.2 mg/ml) and collagenase (0.14 mg/ml) at 37 °C in HBS. The resulting supernatants were collected and centrifuged at 200 × *g* for 5 min. The cells were collected and incubated in MDEM/F-12 medium (GIBCO) containing 5% heat-inactivated horse serum, 0.1 mM ascorbate, insulin-transferring-sodium selenite media supplement (Sigma), 100 U/ml penicillin, 100 μg/ml streptomycin and 0.1 mM bromodeoxyuridine at 37 °C for 1 h. After that, the cells were diluted to 1 × 10^6^ cells/ml and plated in laminin-coated culture dishes. For anoxia/reoxygenation (A/R) treatment, the cells were incubated in an anoxic chamber with a humidified atmosphere of 5% CO_2_ and 95% N_2_ for 30 min. After that, the cells were transferred to a normoxic chamber (95% O_2_ and 5% CO_2_) and incubated for 15, 30, or 60 min.

### Adenoviral vector construction and infection

MiR-33a-5p mimics (mimics-miR-33a-5p), miR-33a-5p inhibitor, and their negative controls (miR-NC and inhibitor-NC, respectively) were designed and synthesized by RiboBio (Guangzhou, China). Small interfering RNAs (siRNAs) targeting USP46 and circ-NNT (si-USP46 and si-circ-NNT, respectively) and a scrambled form used as control (shRNA NC) were obtained from Dharmacon (Lafayette, CO, USA). Mimics-miR-33a-5p, mimics-NC, miR-33a-5p inhibitor, inhibitor-NC, si-USP46, circ-NNT shRNA, and shRNA NC were transfected into cultured cardiomyocytes using Lipofectamine® 2000 (Invitrogen) following manufacturer’s instructions. The circ-NNT exon along with the endogenous flanking sequence (1 kb upstream) was inserted into the pcDNA3.1 vector, and part of the upstream flanking sequence was inserted in an inverted orientation downstream. The mouse USP46 was cloned by PCR using mouse cDNA as the template and inserted into the pcDNA3.1 vector. All vectors (pcDNA-circ-NNT, pcDNA-USP46, and pcDNA3.1 empty vector) as well as si-USP46, circ-NNT shRNA, and shRNA NC were cloned into the Adeno-X Expression System (Clontech, Otsu, Japan) following manufacturer’s instructions. All adenoviral constructs were amplified in HEK293 cells. Adenoviral infection of HEK293 cells or cardiomyocytes was carried out as previously described [39].

### Western blot analysis

Cells or heart tissues were lysed in ice-cold RIPA lysis buffer (Solarbio, Beijing, China) containing protease inhibitors (Roche) for 30 min. Proteins were separated by 12% SDS-PAGE and transferred to nitrocellulose membranes. The membranes were probed with primary antibodies toward caspase-1 (Abcam, Cambridge, UK, ab138483), cleaved caspase-1 (Abcam, ab207802), caspase-11 (Abcam,ab22684), cleaved caspase-11 (Abcam, ab180673), USP46 (Abcam, ab88795), Tubulin (Cell Signaling Technology, Danvers, MA, USA, D65A4), GAPDH (Abcam, ab8245) and β-actin (Cell Signaling Technology, 8H10D10), respectively. After washing with PBS-Tween 20, the membranes were incubated with horseradish peroxidase-conjugated secondary antibodies, and protein bands were visualized using Pierce^®^ ECL Western blotting substrate (Pierce, Rockford, IL, USA). Densitometric analysis was performed using Image J.

### ELISA

USP46, IL-1β, and IL-18 concentrations in serum samples were detected using ELISA kits (Shanghai Westang Biotech, Shanghai, China) following manufacturer’s instructions.

### Real-time PCR (RT-PCR) analysis

Total RNAs from cultured cardiomyocytes or heart tissues were extracted using Trizol reagent (Invitrogen). CDNA synthesis was carried out using the High Capacity cDNA Reverse Transcription Kit (Applied Biosystems, Carlsbad, CA, USA) following manufacturer’s instructions. PCR was performed with the SYBR Green PCR Master Mix Kit (Applied Biosystems). The miR-33a-5p levels were normalized to U6. The USP46 mRNA levels were normalized to Tubulin. The circ-NNT levels and the caspase-1, caspase-11, IL-18, and IL-1β mRNA levels were normalized to β-actin. The relative mRNA expression was calculated using the 2^- ∆ ∆ Ct^ method. The primers used in PCR were:

Circ-NNT: F: 5′-GGAGGCTATGGCACCACTTCA-3′,

 R: 5′-CAGCCAGTAAGCCTGCATTGA-3′;

USP46: F: 5′-CCGAAACATCGCCTCCATCT-3′,

 R: 5′-GTTGAAGAGCCGGAGTTCCA-3′;

Caspase-1: F: 5′-ACACGTCTTGCCCTCATTATCT-3′,

 R: 5′-ATAACCTTGGGCTTGTCTTTCA-3′;

Caspase-11: F: 5′-ATGTGGAGAAGGACTTCATTGC-3′,

 R: 5′-AGATGACAAGAGCAGGCATGTA-3′;

IL-1β: F: 5′-CCCTGCAGCTGGAGAGTGTGG-3′,

 R: 5′-TGTGCTCTGCTTGAGAGGTGCT-3′;

IL-18: F: 5′-ACAACCGCAGTAATACGGAGCA-3′,

 R: 5′-TGTGCTCTGCTTGAGAGGTGCT-3′.

GAPDH: F: 5′-AGAAGGCTGGGGCTCATTTG-3′

 R: 5′-AGGGGCCATCCACAGTCTTC-3′;

U6: F: 5′-GCTTCGGCAGCACATATACTAA-3′,

 R: 5′-AACGCTTCACGAATTTGCGT-3′.

The primers of the 20 circRNAs are in Table 1.

**Table 1:** The sequence for the circRNA primers.

gene	Primer direction	Sequence
hsa_circ_0072816	Forward	5′- AGTGCCAACCTGTGATACCT-3′
	Reverse	5′- CCACCATTACCTCCCACGAG-3′
hsa_circ_0004809	Forward	5′- GGGCACTTGGAGTTTGCTTG-3′
	Reverse	5′- CACCTTCTGGCCAAGGGATA-3′
hsa_circ_0062906	Forward	5′- AGTAACTGAATCCACGAGAGC-3′
	Reverse	5′- TATCCTCCCACTGCCAATCCT-3′
hsa_circ_0058639	Forward	5′- CCGTGAACTACGTGAGGCAA-3′
	Reverse	5′- ACAAGTGATTTCAGTCTGCTGG-3′
hsa_circ_0073474	Forward	5′- GAAGCACTTGCCAGCTCAGA-3′
	Reverse	5′- AGAGAGAAGATCCTTCTGTACAAGC-3′
hsa_circ_0058416	Forward	5′- TCTTTGGTTCCACCCTGAGC-3′
	Reverse	5′- GAAGGAAGCCAGTAGCAGCA-3′
hsa_circ_0031933	Forward	5′- ACACCGAGGCTGAACACTTT-3′
	Reverse	5′- ATGGAAAGCCCACACTCCAG-3′
hsa_circ_0089200	Forward	5′- AGGAAACGACCCCAACATCG-3′
	Reverse	5′- TCGCACTGGAACTCTTTGGG-3′
hsa_circ_0085765	Forward	5′- TAACGGACAAGGGCTGCAA-3′
	Reverse	5′- GCAGGCCACATGCTTTACTTT-3′
hsa_circ_0063551	Forward	5′- GAAAGAAGCCGCCTGTGGA-3′
	Reverse	5′- AGATGGCAGTGGAGGAGGAT-3′
hsa_circ_0051202	Forward	5′- ATCGCCAAGATGCCAGTCAA-3′
	Reverse	5′- ACATCGCTCTTGCTGGTGTA-3′
hsa_circ_0055893	Forward	5′- CTGTCTGGGTGGTGGATGTG-3′
	Reverse	5’- AAAGGGTAAGAGCCCACACG-3′
hsa_circ_0016802	Forward	5′- AGTTTGCTGATCAGGAGATAGTGT-3′
	Reverse	5′- TCACTGTTTGGTCCAGCAACT-3′
hsa_circ_0082734	Forward	5′- CAGGTGTTCCAGAGCGAGTT-3′
	Reverse	5′- TACGGAAAGTGACCCAGCAC-3′
hsa_circ_0020155	Forward	5′- GACTTGGAGGTGAGCAACAGA-3′
	Reverse	5’- GTGTCCTTTTCTGTATAACGTGGCT-3′
hsa_circ_0086100	Forward	5′- GTATGGCGTGGCTCTCAACA-3′
	Reverse	5′- GAGATGAAGGTGGCCTGGG-3′
hsa_circ_0030509	Forward	5′- TGGTGGTCTTGGTCTGTTTGG-3′
	Reverse	5′- ACAGCACAACCAGATTCTCC-3′
hsa_circ_0030835	Forward	5′- TGCACAGCCAGACCATTCAG-3′
	Reverse	5’- TGGCGCACTTCTAAACTCCT-3′
hsa_circ_0024380	Forward	5′- GGCGTTCTGGATCGTCTTCT-3′
	Reverse	5′- GAAGTCTTTGCGAGCGACCA-3′
hsa_circ_0072424	Forward	5′- GGAGGCTATGGCACCACTTCA-3′
	Reverse	5′- CAGCCAGTAAGCCTGCATTGA-3′

### Immunofluorescence analysis

Cells were fixed in 4% paraformaldehyde for 30 min followed by permeabilization with 0.25% Triton X-100 in PBS at room temperature for 10 h. The cells were subsequently incubated with anti-caspase-1 antibody (1:200) overnight at 4 °C. After washing, the cells were incubated with FITC-conjugated anti-rabbit IgG (1:20; Abcam) at 37 °C for 1 h, counterstained with DAPI (4′, 6-diamidino-2-phenylindole) and subjected to microscopic analysis under a confocal laser scanning microscope (Olympus, Tokyo, Japan).

### Histological analysis

Histological analysis was carried out as we previously described [40]. Briefly, hearts were excised, fixed in 10% formalin, embedded in paraffin and sectioned into 4-μm slices. The slides were stained with hematoxylin-eosin (H&E) and examined under a light microscope. The cross-sectional area of cardiomyocytes was determined by staining with FITC-conjugated wheat germ agglutinin (Sigma). Cardiac fibrosis was assessed by standard Masson trichrome staining (Sigma) following manufacturer’s instructions.

### Luciferase reporter assay

Mouse USP46 3′-UTR (USP46-WT) was amplified by PCR using the following primers: forward, 5′-CCGAAACATCGCCTCCATCT-3′; reverse, 5′-GTTGAAGAGCCGGAGTTCCA-3′. A mutant USP46 3′-UTR derivative containing mutations at the putative miR-33a-5p binding site (USP46-Mut) was created using the QuikChange II XL Site-Directed Mutagenesis Kit (Stratagene). Mouse circ-NNT (circ-NNT-WT) was amplified by PCR using the following primers: forward, 5’-GGAGGCTATGGCACCACTTCA-3′; reverse, 5′-CAGCCAGTAAGCCTGCATTGA-3′. HEK293 cells or cardiomyocytes were first transfected with mimics-miR-33a-5p or mimics-NC, and then with the indicated luciferase construct using Lipofectamine 2000 according to manufacturer’s instructions. Luciferase activity was determined 48 h after transfection and normalized to Renilla activity.

### AGO2 immunoprecipitation

Cultured cardiomyocytes were transfected with miR-33a-5p (miR-33a-5p-WT) or miR-33a-5p mutant (miR-33a-5p-Mut) for 48 h. The cells were lysed in lysis buffer (150 mM KCl, 25 mM Tris-HCl, 5 mM EDTA, 0.5% Triton X-100, 5 mM dithiothreitol (DTT), pH 7.4) supplemented with Ribolock (Fermentas) and proteinase inhibitor cocktail (Roche). The lysates were mixed with anti-AGO2 antibody or IgG-coated sepharose beads and incubated at 4 °C under rotation for 4 h. The beads were subsequently washed six times in lysis buffer, and the RNA was extracted using Trizol reagent (Invitrogen). Circ-NNT and GAPDH mRNA levels were determined by RT-PCR. The immunoprecipitate (IP) to input ratios were calculated.

### Pull-down assay with biotinylated DNA probe

The DNA probe (GGAAATCAACCTTGACAATGCAATT) complementary to circ-NNT was biotinylated. Streptavidin-coated magnetic beads (Sigma) were incubated with the biotinylated circ-NNT probe or a biotinylated random probe (CATGTGTGTGGGTGTGACTTTGACAGCTGCTATTGGGGGTGCTGACATGCCCGTCGTTAT) in wash/binding buffer (0.5 M NaCl, 20 mM Tris-HCl, 1 mM EDTA, pH 7.5) at 25 °C for 2 h to generate probe-coated beads. Cardiomyocytes lysates were incubated with probe-coated beads, and the RNAs bound to the beads were extracted. MiR-33a-5p levels were determined by Northern blot analysis.

### Pull-down assay with biotinylated miRNA

Cardiomyocytes were transfected with biotinylated miR-33a-5p (Bio-miR-33a-5p-WT), biotinylated miR-33a-5p mutant (Bio-miR-33a-5p-Mut) or a biotinylated miRNA with a random sequence (Bio-NC) for 72 h. After washing, the cells were lysed in lysis buffer (20 mM Tris, pH 7.5, 200 mM NaCl, 2.5 mM MgCl2, 0.05% Igepal, 60 U/ml Superase-In (Ambion), 1 mM DTT, protease inhibitor cocktail (Roche)) on ice for 10 min. The lysates were precleared by centrifugation and incubated with M-280 streptavidin-coated magnetic beads (Sigma) at 4 °C for 3 h. To prevent non-specific binding of RNAs and proteins, the beads were pre-coated with RNase-free BSA and yeast tRNA (both from Sigma). After incubation, the beads were washed twice with ice-cold lysis buffer, three times with low salt buffer (0.1% SDS, 1% Triton X-100, 2 mM EDTA, 20 mM Tris-HCl, pH 8.0, 150 mM NaCl) and once with high salt buffer (0.1% SDS, 1% Triton X-100, 2 mM EDTA, 20 mM Tris-HCl, pH 8.0, 500 mM NaCl). The RNAs bound to the beads were extracted, and circ-NNT levels were analyzed by RT-PCR.

### Northern blot analysis

Cardiomyocytes lysates were incubated with circ-NNT probe or random probe-coated magnetic beads. After washing, the RNAs bound to the beads were extracted, separated on a 15% polyacrylamide-urea gel, transferred to positively charged nylon membranes (Millipore) and immobilized through covalent linkage to the membrane by UV irradiation. The membranes were subjected to hybridization with 100 pmol 3′-digoxigenin (DIG)-labelled probes overnight at 42 °C. Probes were labelled with DIG using a 30-End DIG Labelling Kit (Roche). The signals were detected using a DIG luminescent detection kit (MyLab) following manufacturer’s instructions.

RNase R treatment was carried out using 2 μg total RNA treated with or without RNase R (2.5 U/μg, Epicentre) for 20 min at 37 °C. RNA was phenolised, ethanol-precipitated, and 20% were used for RT-PCR.

### Animal model and treatment

Male adult C57BL/6 mice (8 weeks old) were obtained from Minhang Central Hospital. All the animal experiments were made in the animal laboratory in Minhang Hospital and the animal studies were conducted in accordance with the National Institutes of Health guidelines for the Care and Use of Minhang Hospital. To assess myocardial I/R injury, mice were subjected to 45 min myocardial ischemia followed by 60, 120, and 180 min reperfusion as previously described [41]. Sham-operated group underwent the same procedure except that the snare was left untied. After reperfusion, evans blue dye (1 ml of a 2% solution; Sigma-Aldrich) was injected through jugular vein to delineate the ischemic area at risk. The mice were euthanized by cervical dislocation. Then the heart was rapidly excised and sectioned. The heart slices were incubated in 1.0% 2, 3, 5-triphenyltetrazolium chloride (TTC; Sigma-Aldrich) for 15 min at 37 °C to differentiate live (red) from dead or infarcted myocardium (white). After washing in ice-cold sterile saline, the slices were fixed in 10% formaldehyde, weighed and photographed from both sides. The infarct area (INF) and the risk zone were assessed using computer-assisted planimetry by a histologist blinded to treatment conditions. The INF/LV ratio (%) and the infarct size (defined as % of risk zone) were calculated.

For miRNA delivery, mimics-miR-33a-5p or mimics-NC was administered by intravenous injection at a dose of 30 mg/kg per day for three consecutive days. The mice were then subjected to I/R treatment. For intracoronary delivery of adenoviruses, adenoviruses carrying pcDNA-USP46 (200:1 m.o.i.), pcDNA empty vector (250: 1 m.o.i.), si-USP46 (200:1 m.o.i.), circ-NNT shRNA (250:1 m.o.i.) or shRNA NC (250:1 m.o.i.) were injected with a catheter from the LV apex into the aortic root as previously described [41]. The mice were subjected to I/R treatment five days after injection of adenoviruses.

### Assessment of cardiac function by echocardiography

Echocardiographic examination was performed using the Vevo 770 high-resolution echocardio-garpgic system (Visual Sonics Inc., Toronto, ON, Canada) at 2, 4, and 6 weeks after myocardial IRI. The experimental groups are as follows: (i) I/R, I/R + shRNA NC, I/R + circ-NNT; (ii) I/R, I/R + si-NC, I/R + si-USP46. Six mice in each group were anaesthetized with 100 mg/kg ketamine and 20 mg/kg xylazine and placed in a supine position. Then Mice were subjected to 45 min myocardial ischemia followed by 180 min reperfusion, after that, cardiomyocytes transfected with shRNA-NC/circ-NNT shRNA or si-NC/si-USP46 were intravenous injected into mice At 2, 4, or 6 weeks, M-mode tracings were recorded from a parasternal short-axis view and functional systolic and diastolic parameters were obtained. Ejection fraction (EF) and fractional shortening (FS) were calculated from digital images using Vevo 770 software.

### Microarray processing

Global profiling of mice circRNAs was performed using an Arraystar circRNA Microarray, version 2.0 (Arraystar Inc., Rockville, MD, USA). Mice were subjected to 45 min myocardial ischemia followed by 180 min reperfusion, sham-operated group underwent the same procedure except that the snare was left untied. Briefly, total RNA was digested with RNase R (Epicentre, Inc., Madison, WI, USA) to remove linear RNAs. The enriched circRNAs were then amplified and hybridized. circRNAs were differentially expressed between the two samples were identified by fold-change filtering. Hierarchical clustering was used to reveal distinguishable circRNA expression pattern in samples.

### Statistical analysis

All results are expressed as the means ± standard deviation (SD). Data were interpreted using IBM SPSS 13.0 (SPSS Inc., Chicago, IL, USA). Statistical analysis was performed using Student’s *t* test. One-way analysis of variance (ANOVA) was employed for multiple comparisons. *P* values <0.05 indicated a statistically significant difference.

## Supplementary information

Supplementary materials

Supplementary Figure S1

Supplementary Figure S2

Supplementary Figure S3

Supplementary Figure S4

Supplementary Figure S5

Attribution of Authorship

## Data Availability

The datasets used and/or analyzed during the current study are available from the corresponding author on reasonable request.
