# Supplementary material for: CircRNA circ-NNT mediates myocardial ischemia/reperfusion injury through activating pyroptosis by sponging miR-33a-5p and regulating USP46 expression
Source: Cell Death Discov. 2021 Nov 29;7:370. doi: 10.1038/s41420-021-00706-7 (PMC8630116; doi:10.1038/s41420-021-00706-7)
Supplement: Supplementary file 1 — Supplementary materials [file 41420_2021_706_MOESM1_ESM.doc]

**Supplementary Figure Legends**

**Figure S1**. Pyroptosis is activated following myocardial I/R *in vivo* and in cultured cardiomyocytes following A/R *in vitro*. (A-D) C57BL/6 mice were subjected to 45 min myocardial ischemia followed by 60, 120 and 180 min reperfusion as indicated. Sham group were included as control. Relative mRNA levels of pro-caspase-1 and cleaved caspase-1 (A), pro-caspase-11 and cleaved caspase-11 (B), IL-1β (C) and IL-18 (D) in heart tissues were detected by RT-PCR. *n*=6/group. (E-H) *In vitro*, Cardiomyocytes were subjected to 30 min anoxia followed by 0, 15, 30 and 60 min reoxygenation. Relative mRNA levels of pro-caspase-1 and cleaved caspase-1 (E), pro-caspase-11 and cleaved caspase-11(F), IL-1β (G) and IL-18 (H) were detected by RT-PCR. n = 3, *P < 0.05, ***P* < 0.01, ****P* < 0.001.

**Figure S2.** The expression of circ-NNT in different tissues was examined in I/R mice.

**Figure S3. C**haracterization of circ-NNT. **(A)** UCSC database showed that NNT was a nearby gene for circ-NNT (hsa_circ_NNT). (B) Full length of circ-NNT (hsa_circ_0072424) was conversed in mammals.

**Figure S4**. Circ-NNT is induced by I/R and mediates myocardial I/R injury *in vivo* and A/R-stimulated pyroptosis in cardiomyocytes. (A) Mice received adenoviruses carrying si-circ-NNT or si-NC by injection as described in methods. After 5 days, the mice were subjected to myocardial I/R (45 min/180 min). Relative circ-NNT levels in cardiac tissues were detected by RT-PCR. *n*=6/group. (B) Relative circ-NNT expression in cardiomyocytes transfected with circ-NNT shRNA or shRNA-NC as indicated by determined by RT-PCR. *n*=3. (C-D) Cardiomyocytes transfected with circ-NNT shRNA or shRNA-NC were subjected to A/R (30 min/60 min) as indicated. Untransfected cells were included as control. (C) Relative circ-NNT levels were determined by RT-PCR. *n*=3. (D)Caspase-1 was detected by immunofluorescence staining (magnification x200). Cells were stained with DAPI. **P*<0.05, ***P*<0.01.

**Figure S5.** H&E staining results of myocardial tissues at 2, 4 and 6 weeks. (A) H&E staining results of myocardial tissues of I/R, I/R+ shRNA-NC and I/R+circ-NNT shRNA groups at 2, 4 and 6 weeks. (B) H&E staining results of myocardial tissues of I/R, I/R+ si-NC and I/R+si-USP46 groups at 2, 4 and 6 weeks.
