# Supplementary figures and images for: CircRNA circ-NNT mediates myocardial ischemia/reperfusion injury through activating pyroptosis by sponging miR-33a-5p and regulating USP46 expression
Source: Cell Death Discov. 2021 Nov 29;7:370. doi: 10.1038/s41420-021-00706-7 (PMC8630116; doi:10.1038/s41420-021-00706-7)

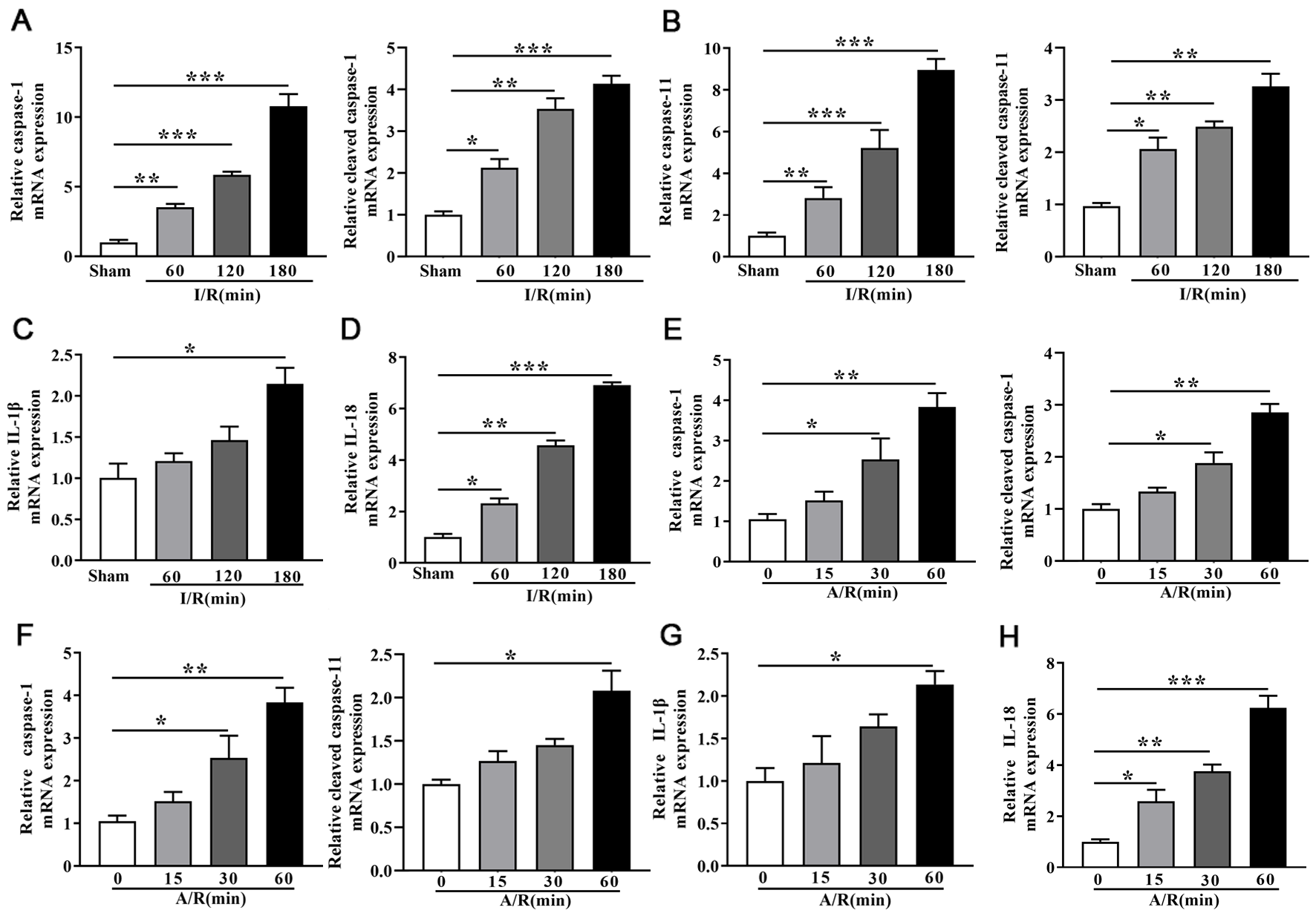

Supplement: Supplementary file 2 — Supplementary Figure S1 [file 41420_2021_706_MOESM2_ESM.tif]

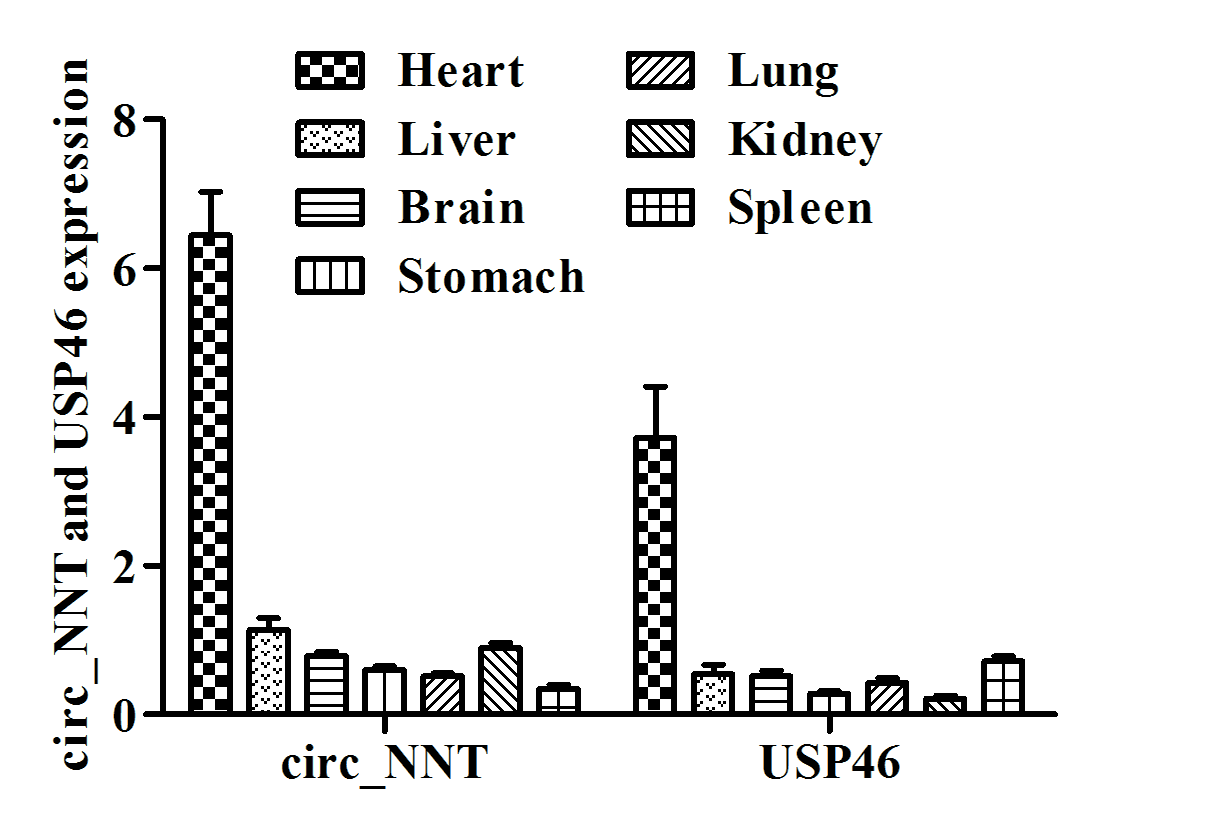

Supplement: Supplementary file 3 — Supplementary Figure S2 [file 41420_2021_706_MOESM3_ESM.tif]

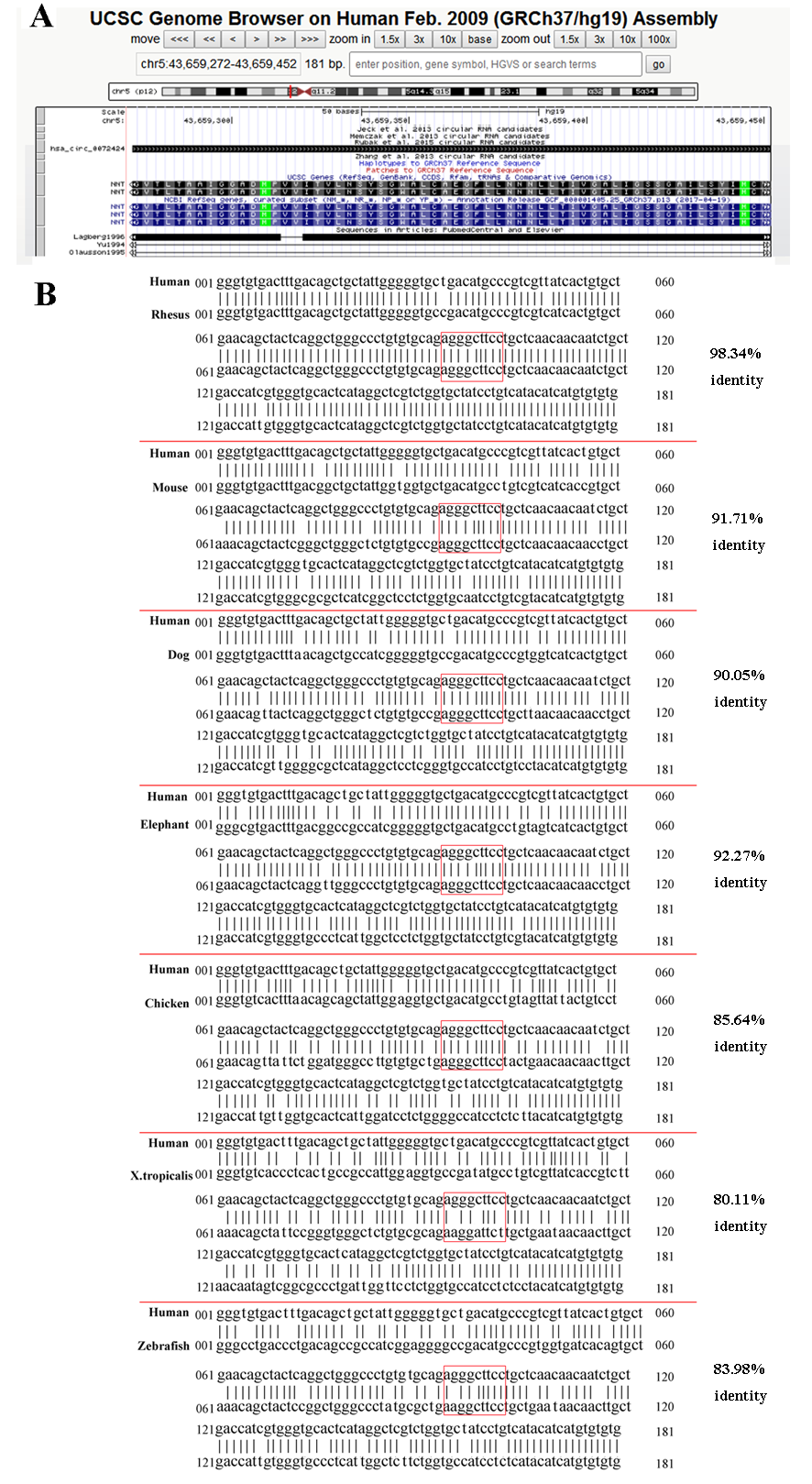

Supplement: Supplementary file 4 — Supplementary Figure S3 [file 41420_2021_706_MOESM4_ESM.tif]

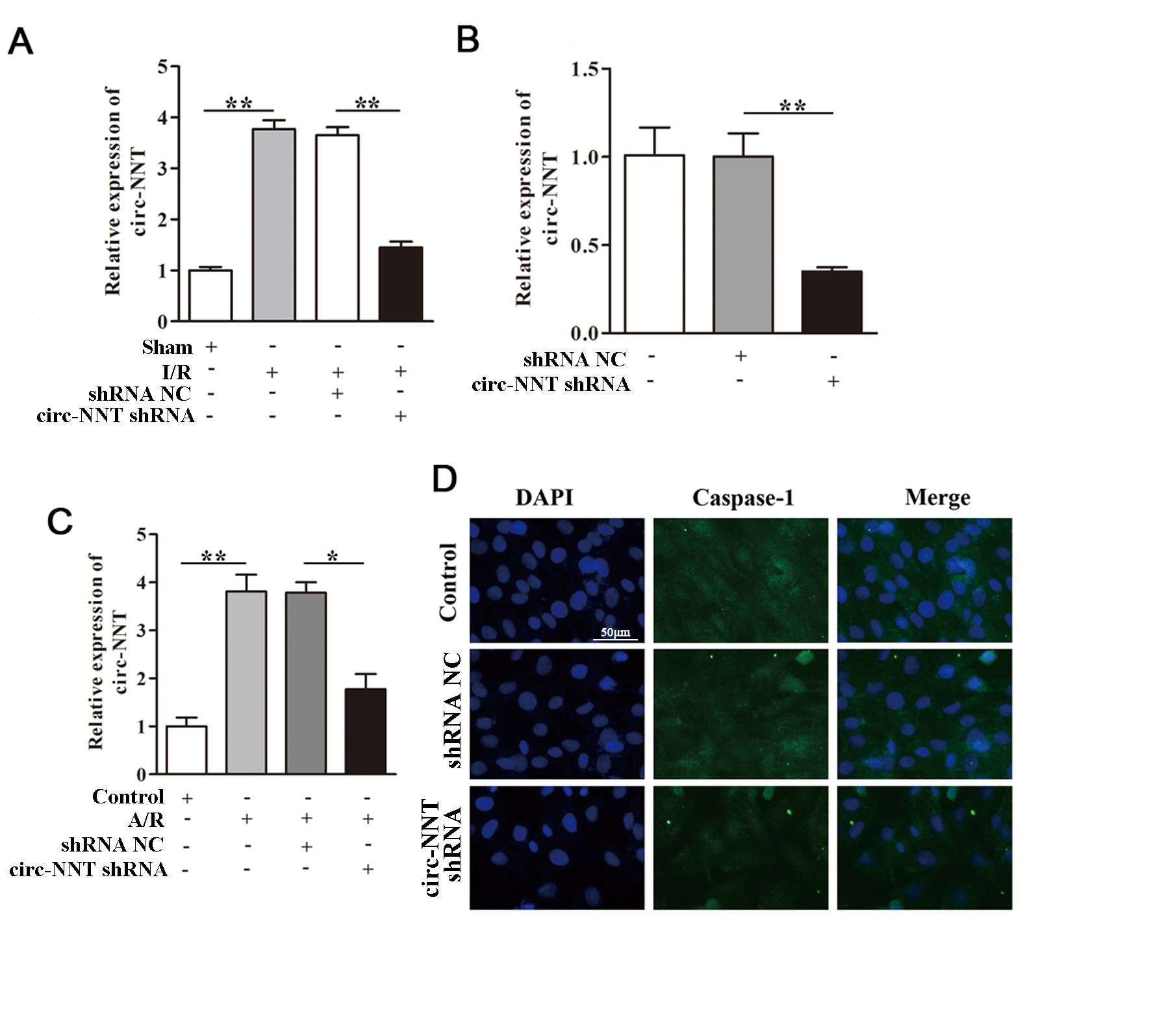

Supplement: Supplementary file 5 — Supplementary Figure S4 [file 41420_2021_706_MOESM5_ESM.tif]

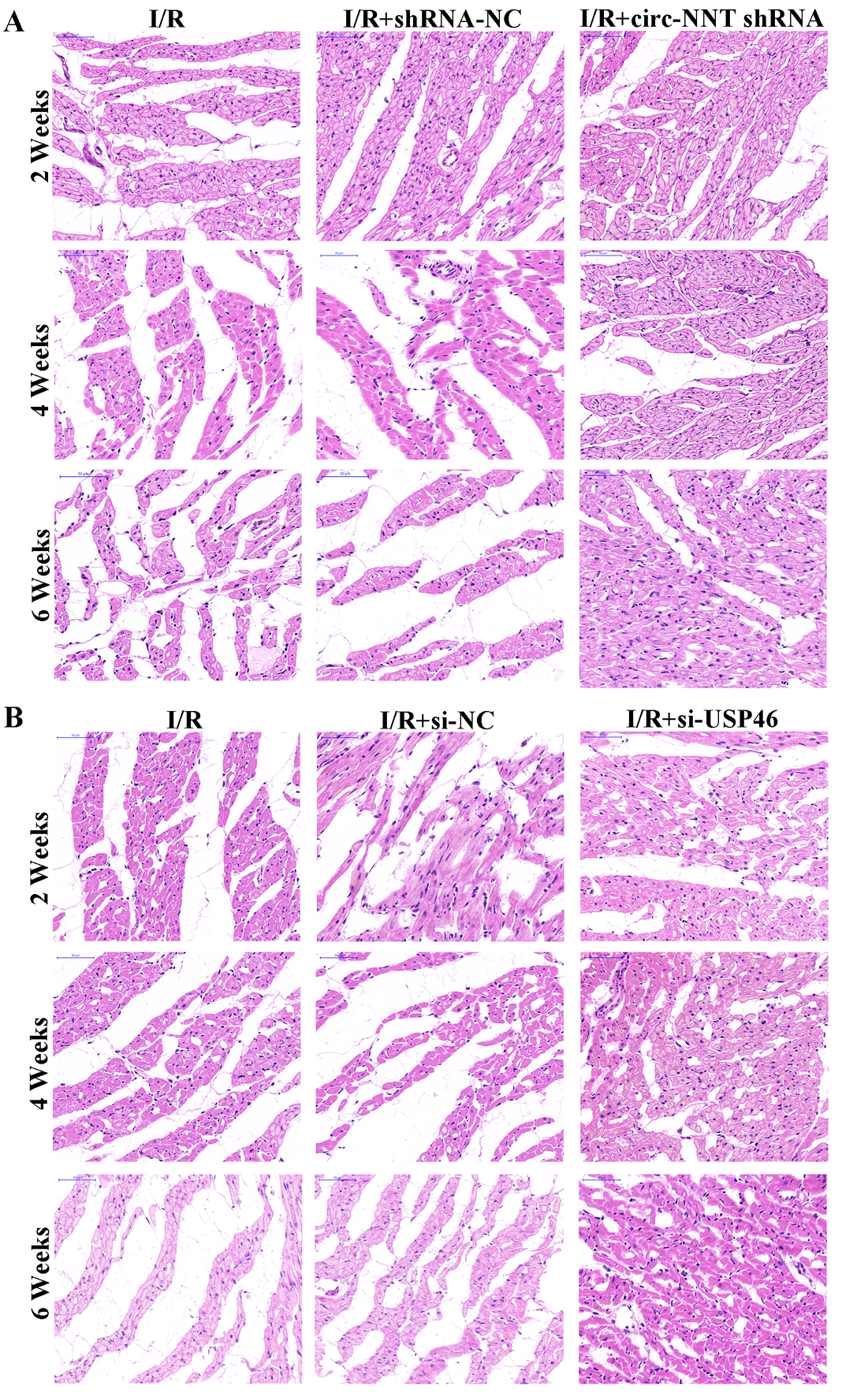

Supplement: Supplementary file 6 — Supplementary Figure S5 [file 41420_2021_706_MOESM6_ESM.tif]
