# Supplementary material for: CircRNA circ-NNT mediates myocardial ischemia/reperfusion injury through activating pyroptosis by sponging miR-33a-5p and regulating USP46 expression
Source: Cell Death Discov. 2021 Nov 29;7:370. doi: 10.1038/s41420-021-00706-7 (PMC8630116; doi:10.1038/s41420-021-00706-7)
Supplement: Supplementary file 7 — Attribution of Authorship [file 41420_2021_706_MOESM7_ESM.pdf]

DECLARATION OF CONTRIBUTIONS TO ARTICLE

ADMC

Manuscript Number:	Journal Name:	
CDDISCOVERY-21-2031	*Cell Death Discovery*	(the 'Journal')

Proposed Title of the Contribution:

CircRNA circ-NNT mediates myocardial ischemia/reperfusion injury through activating pyroptosis by sponging miR-33a-5p and regulating USP46 expression (the 'Contribution)

Author(s):

Xiaomiao Ye, Yanwen Hang, Yi Lu, Dandan Li, Fangfang Shen, Ping Guan, Jian Dong, Ludong Shi, Wei Hu (the 'Authors')

For all *CDDis*covery articles, each person named as an author in the published version must be able to show he or she has contributed substantially to the article.

Authorship credit should be based on 1) substantial contributions to conception and design, acquisition of data, or analysis and interpretation of data; 2) drafting the article or revising it critically for important intellectual content; and 3) final approval of the version to be published. Authors should meet conditions 1, 2 and 3.

Any person who cannot be shown to have made a substantial contribution to the article cannot be listed as an author in the final version. The name of any person who is deemed to have made a minor contribution can, however, appear in the Acknowledgments section of the article.

Please complete the table below to indicate the contributions of all named authors to the manuscript.

Author Full Name:	Specification of Contribution to the Manuscript:
Xiaomiao Ye	Acquisition of data, analysis and interpretation of data, manuscript preparation and final approval of manuscript
Yanwen Hang	Acquisition of data, analysis and interpretation of data, manuscript preparation and final approval of manuscript
Yi Lu	Acquisition of data, analysis and interpretation of data and final approval of manuscript
Dandan Li	Acquisition of data, analysis and interpretation of data and final approval of manuscript
Fangfang Shen	analysis and interpretation of data and final approval of manuscript
Ping Guan	analysis and interpretation of data and final approval of manuscript
Jian Dong	analysis and interpretation of data and final approval of manuscript
Ludong Shi	Substaintial contributions to conception and design the manuscript and final approval of manuscript
Wei Hu	Substaintial contributions to conception and design the manuscript and final approval of manuscript

Please complete the table below to indicate the contributions of all named authors to the figures.

Figure 1:

acquisition of data: Xiaomiao Ye,Yi Lu
analysis of data: Xiaomiao Ye,Yi Lu,Fangfang Shen,Ping Guan
interpretation of data: Xiaomiao Ye,Yi Lu,Dandan Li
assembled the figure: Xiaomiao Ye

Figure 2:

acquisition of data: Xiaomiao Ye,Yi Lu
analysis of data: Xiaomiao Ye,Yi Lu,Fangfang Shen,Ping Guan
interpretation of data: Xiaomiao Ye,Yi Lu,Dandan Li
assembled the figure: Xiaomiao Ye

Figure 3:

acquisition of data: Xiaomiao Ye,Dandan Li
analysis of data: Xiaomiao Ye,Dandan Li,Fangfang Shen,Jian Dong
interpretation of data: Xiaomiao Ye,Dandan Li,Fangfang Shen
assembled the figure: Xiaomiao Ye

Figure 4:

acquisition of data: Yanwen Hang,Yi Lu
analysis of data: Yanwen Hang,Yi Lu,Fangfang Shen,Jian Dong
interpretation of data: Yanwen Hang,Yi Lu,Fangfang Shen
assembled the figure: Yanwen Hang

Figure 5:

acquisition of data: Yanwen Hang,Yi Lu
analysis of data: Yanwen Hang,Yi Lu,Ping Guan
interpretation of data: Yanwen Hang,Yi Lu,Dandan Li
assembled the figure: Yanwen Hang

Figure 6:

acquisition of data: Yanwen Hang,Dandan Li
analysis of data: Yanwen Hang,Dandan Li,Ping Guan
interpretation of data: Yanwen Hang,Dandan Li,Ping Guan
assembled the figure: Yanwen Hang

Signed for and on behalf of the Author(s):	Print Name:	Date:
Wei hu	Wei Hu	2021.08.23
